# A study of the effect of a visual arts-based program on the scores of Jefferson scale for physician empathy

**DOI:** 10.1186/1472-6920-13-142

**Published:** 2013-10-25

**Authors:** Kuang-Tao Yang, Jen-Hung Yang

**Affiliations:** 1 Center for Medical Humanities and Department of Nuclear Medicine, Changhua Christian Hospital, Changhua 50012, Taiwan; 2 School of Medicine, Tzu Chi University, and Department of Dermatology, Tzu Chi General Hospital, Hualien 97004, Taiwan

**Keywords:** Visual arts, Jefferson scale for physician empathy, Empathy, Medical humanities, ACGME

## Abstract

**Background:**

The effect of visual arts interventions on development of empathy has not been quantitatively investigated. A study was conducted on the effect of a visual arts-based program on the scores of the Jefferson Scale for Physician Empathy (JSPE).

**Methods:**

A total of 110 clerks (n = 92) and first-year postgraduate residents (PGY1s) (n = 18) participating in the program were recruited into this study. The 4-hr program covered the subjects of learning to interpret paintings, interpreting paintings relating to medicine, illness and human suffering, the related-topics of humanitarianism and the other humanities fields and values and meaning. The JSPE was completed at the beginning (pretest) and the end (posttest) of the program.

**Results:**

There was no significant difference between the pretest and posttest JSPE scores. The average of the scores for the pretest was lower in the subgroup of PGY1s than the subgroup of clerks (*p* = 0.0358). An increased but not significantly mean posttest JESPE score was noted for the subgroup of PGY1s. Neither the females nor the males had higher posttest JSPE scores than the pretest scores.

**Conclusions:**

Although using a structured visual arts-based program as an intervention may be useful to enhance medical students’ empathy, our results failed to show a positive effect on the JSPE Scores for a group of clerks and PGY1s. This suggests that further experimental studies are needed if quantitative evaluation of the effectiveness of visual-arts based programs on empathy is to be investigated.

## Background

The role of the humanities including visual arts, literature and many other fields has been highlighted in many publications [1,2]. A literature review demonstrated that there was a need for further studies to evaluate art-based courses in terms of their effects in medical education [3]. Several studies have investigated the effectiveness of arts-based interventions in improving observation skills [1,4,5]. But, the effect of visual arts-based interventions on the development of empathy has not been extensively studied. There were limited qualitative studies in this aspect. Reilly et al. reported that through the analysis of paintings, medical students had been shown to increase empathy and sensitivity to the art of medicine [4]. In a qualitative study, we have reported that a structured visual-arts based program might be helpful to enhance students’ empathy [6].

The *Jefferson Scale of Physician Empathy* (JSPE) is a validated instrument designed to measure empathy specific to health professionals (HP version) and medical students (S version) [7]. In a cross-sectional study of American medical students, first-year students were observed to have obtained the highest JSPE scores while fourth-year students scored the lowest [8], although reports of the decline of empathy during medical education were criticized to be exaggerated [9]. It is interesting to know whether art-based interventions will be able to have an impact on JSPE scores, especially for the students entering clinical courses and the first-year postgraduate residents (PGY1s) entering clinical practice. We conducted a study on the effect of the visual arts-based program on the scores of JSPE.

## Methods

### Participants

Our hospital (Changhua Christian Hospital) is a 1,600-bed medical center and is one of the main teaching hospitals in central Taiwan. In this country, there are 7-year (applicants are high school graduates) and 5-year graduate medical programs. Some programs integrate Chinese medicine courses. After 2–4 years of pre-medical and basic sciences, medical students require 2 years of clerkship and 1 year of internship before graduation. Recruitment of participants in this study was through convenience sampling. From 2010 October to 2011 January, all of 98 medical students in clerkship and 22 PGY1s at our hospital participated in a required visual arts-based program. Among them 113 (95 clerks and 18 PGY1s) were recruited into this study. The participants were divided into 12 discussion small groups so that there were less than 12 persons for each group. Those already in the same block rotations were allocated into one small group when possible. The complexity of different rotations and duty loads of clinical care made some participants have to shift to join one of the pretest or posttest sessions in the other groups. There were 3 of the recruited unable to complete the 2-h sessions within 1 week as planned. The data were excluded from analysis. To this end, there was a total of 110 (92 clerks and 18 PGY1s) recruited participants in 10 groups, that completed the study. Ninety of the 92 clerks were the year 6 students of 7-year medical programs. Only 2 of 92 were the year 4 students of 5-year graduate medical programs. All PGY1s were graduates of 7-year program. Data from these 110 students were used for analysis of the effect of the program on the JSPE scores.

### Visual arts-based program

We implemented a visual arts-based program as an intervention. The program was initially initiated with the goals of giving medical students entering their clinical training in our hospital an appreciation of paintings, helping them use visual arts as a tool to develop competencies as physicians and interesting them in visual arts [6]. It was a required core course conducted for medical students in clerkship and internship in our hospital from 2008 December to 2010 May. It was required then for the PGY1s who had not ever participated in the program. In brief, an approach was used with a structured program. The program was 4 hours long and had small discussion groups (7–12 persons each group). It was subdivided into steps including using stories, histories and images to increase motivation, learning to interpret paintings, interpreting medicine-related paintings, interpreting paintings relating to human suffering and relating to humanitarianism, discussing related-topics in the other humanities fields (such as novels, poems, musicals and films) and discussing the values and meaning. Students were asked to describe their opinions about the paintings relating to disease and human suffering and relate to humanitarianism and the related-topics in the other humanities fields (examples: the compassionate connections with patients of a physician in a clip from the film “Patch Adams” and the theme of the social and spiritual isolation of the transformed hero in the short novel “The Metamorphosis” by Franz Kafka). The rationale was to take holistic care into consideration. Physicians must seek to understand in the context of patients’ beliefs, and family and cultural values [10] and the personal health care should include physical, mental, emotional and social concerns [11]. The contents and subjects of our program also encompassed the six realms of meaning suggested by Phenix [12] and the five topic groups in medical humanities proposed by Evans [13]. The attendees were enthusiastic in discussion and response during the program in our previous study. The average rating of participants’ feedback on anonymous questionnaires for the question of “I enjoyed the program” was 4.3 on a scale of 1–5. Content analysis of the responses from participating students revealed that the course was interesting and was helpful to enhance empathy and other related skills such as listening and communication and learning to build teamwork [6]. These skills are needed for competencies suggested by the Accreditation Council for Graduate Medical Education (ACGME).

### JSPE

As the JSPE was developed to specifically measure the empathy in medical professionals and students, the JSPE (student version) was used in this study. In the questionnaire, there were twenty items to be answered with a seven-point Likert scale (1 = strongly agreed to 7 = strongly disagreed). Among them, ten were negatively worded items, which were to be reversely scored. A higher score indicates a higher level of empathy. A sample of positively worded question is: “I believe that empathy is an important therapeutic factor in medical treatment” and a sample of negatively worded question is “It is difficult for a physician to view things from patients’ perspectives.”

The JSPE has been translated into about 25 languages. The version used in this study was translated into Chinese and then back-translated into English by two bilingual translators to ensure the accuracy of translation. The psychometric characteristics of the translated version needed to be defined. Data collected from the study were subjected to exploratory factor analysis (maximum likelihood) with Varimax rotation. In order to expand the volume of the data, those JSPE scores collected at the beginning of the first session of the studies from all of 113 recruited were analyzed. Three factors retained using Velicer’s minimum average partial criteria [14]. The first factor accounted for 28% of the total variance and was similar to the grand factor of “perspective taking” (21% of the variance) in Hojat’s original study [7]. The second factor and the third factor accounted to 11% and 8% of the variance and were similar to the second factor (compassionate care; 8% of the variance) and the third factor (to stand in patient’s shoes; 7% of the variance) in Hojat’s study, respectively. The internal consistency reliability evaluated using the Cronbach coefficient alpha was 0.81. The mean was 110.92 ± 10.3 with a range of 86–140.

### Procedures

In this study, the 4-hr program was divided into two 2-hr sessions (each one week apart). Both recruited and not recruited might attend the same group. The (pretest) JSPE with another anonymous questionnaire for program assessment were distributed at the beginning of the first session. No limitation was for the time to complete the JSPE. At the end of the program, the (posttest) JSPE were distributed again with another anonymous questionnaire for post-lecture program assessment. Students recruited were asked to complete both while not recruited to complete at least the program assessment. Those not recruited were informed that they may complete the JSPE but their scores would not be included in the analysis. The pretest and posttest JSPE scores collected were used for analysis.

### Ethic aspects

The hospital’s Educational Board approved the program and the Institutional Review Board of the hospital approved the study.

### Statistics

Paired t-test was used to test the significant difference of the JSPE scores between the pretest and posttest. Unpaired t-test was used to test the significant difference of the JSPE scores between the subgroups of PGY1s and clerks.

## Results

One hundred and ten participants (92 clerks and 18 PGY1s; 33 females and 77 males) completed both sessions of the program. The distribution of the participants and the results of the pretest and posttest JSPE scores are shown in Table 1. No significant difference was observed between the pretest and the posttest JSPE scores. There was no significant difference between the pretest and the posttest scores for the subgroup of PGY1s and clerks, respectively. The average of the score for the pretest was lower in the subgroup of PGY1s than the subgroup of clerks (*p* = 0.0358) with a Cohen’s d = 0.553. Significant difference was not noted for the posttest scores between the subgroups of PGY1s and clerks. In terms of gender, either the females or the males did not have a higher posttest JSPE scores than the pretest. No difference between the pretest and posttest for the females or males in the subgroup of clerks, respectively. Neither was it for the subgroup of or PGY1s.

**Table 1:** Results of the pretest and posttest JSPE scores

	**Pretest**		**Posttest**	
	**No**	**Mean ± SD**	**No**	**Mean ± SD**
**Total**	110	110.92 ± 10.27	110	111.30 ± 11.57
**Gender**				
Female	33	112.36 ± 10.52	33	114.36 ± 11.20
Male	77	110.29 ± 10.17	77	109.99 ± 11.54
**Training level**				
Clerk*	92	111.82 ± 10.22	92	111.42 ± 11.70
Female	29	112.41 ± 11.00	29	114.90 ± 11.05
Male	63	111.48 ± 10.03	63	109.81 ± 11.72
PGY1	18	106.28 ± 9.50	18	110.72 ± 11.18
Female	4	113.00 ± 8.08	4	110.50 ± 13.30
Male	14	104.64 ± 9.67	14	110.79 ± 11.07

*Significant difference (*p = 0.0358*) between the subgroups of clerks and PGY1s for the pretest scores.

## Discussion

Empathy is a cognitive attribute that involves an understanding of the inner experiences and perspectives of the patient, combined with a capability to communicate this understanding to the patient [15]. It is essential in the physician-patient relationship. It has been stated that the medical humanities in general, and reflective writing in particular, have shown empirical promise in terms of helping students to become more aware of emotions and their role in medicine and to express multidimensional empathy [16]. Quantitative evaluation of effects of literature on empathy has been reported. Shapiro et al. used the Empathy Construct Rating Scale and the Balanced Emotional Empathy Scale to evaluate an elective literature course (8-hr over 4 months) to teach empathy to first-year medical students [17]. Sands et al. used the Interpersonal Reactivity Index to evaluate the effect of narrative medicine on empathy for the members of a pediatric oncology team (weekly training seminar for 6 weeks) [18]. In one report, most of the students made significant gains in their ability to make empathetic responses after participation in communication skills training (two 1.5-h workshops, a week apart) [19]. Perry et al. suggested that study of the arts could directly provide students with stimulation of the wider experience of life necessary for mature interaction with other human beings and participation in the artistic process might help students to explore their own feelings, question them and develop new ways of thinking. Both can help to enhance empathy [3]. Discussions on paintings relating to human disease, suffering and the related-topics in the other humanities fields as in our program may be expected to have the participants aware of the need to be respectful to others. However, no statistically significant increase in the JSPE score of the participants was observed after the intervention with a program such as ours in this study. The pretest JSPE for the subgroup of PGY1s was lower significantly than the clerks. The posttest score for PGY1s increased to about the same level of the posttest scores for the subgroup of the clerks. The posttest scores for the PGY1s did not differ significantly from the pretest scores though, probably because of the small sample size. The fact that a large proportion (84%) of the recruited was medical students may be another reason for the negative results of the posttest scores. Because of the relatively modest clinical rotation, there may be lack of the wider experience of interaction with patients and thus less of stimulation for enhancement of empathy for the clerks.

Female medical students were reported to have higher JSPE scores than male in a previous study [8]. It was not remarkable in our study. The smaller proportion of the female group (female to male ratio: 30% to 70%) might partially account for the discrepancy. Shapiro et al. reported that females had significantly more empathy posttest using an elective literature course as intervention [17]. No similar result using our program as intervention was observed. Other than the smaller sample size of the females in our study and the different nature of the influences using literature and visual arts as interventions, the drawback that time for each topic mentioned in this 4-hr program was limited may be another possible reason for our negative result. This may also be the reason for the negative results between our pretest and posttest JSPE scores in this study. Most of the studies quantitatively investigating the effect of humanities on empathy were with the courses in a format of weekly or longer in months [17,18]. The 4-hr was found suitable to complete the steps and the contents of our program based on the previous study. It is reasonable to extend the time of the program and to increase the frequency of the interventions. The participants can be immersed more in the related topics and provided with more stimulation. Addition of more elements from other fields of medical humanities (narrative medicine, drama, literature, films, music and others) to the program may also be possible and useful.

To our knowledge, no previous research has been reported using a validated measurement tool to quantitatively evaluate the effect of a visual arts program on empathy. Whether enhancement of empathy score using such interventions is possible for the medical students and residents and more remarkable for one training level than the others and for females than males is interesting to know. Further experimental studies with larger sample size are needed to clarify this variance. Intervention of such a program with extension of time or increase of the frequency of interventions may also be worthy of studying.

## Conclusions

Although using a structured visual arts-based program as an intervention may be useful to enhance medical students’ empathy, our results failed to show a positive effect on the JSPE Scores for a group of clerks and PGY1s. This suggests that further experimental studies are needed if quantitative evaluation of the effectiveness of visual-arts based programs on empathy is to be investigated.

## Competing interests

The author declares that they have no financial or non-financial competing interests.

## Author’s contributions

The author contributed to the conception, design and implementation of the program and drafted the manuscript. Both authors read and approved the final manuscript.

## Pre-publication history

The pre-publication history for this paper can be accessed here:

http://www.biomedcentral.com/1472-6920/13/142/prepub
